# A Single Subset of Dendritic Cells Controls the Cytokine Bias of Natural Killer T Cell Responses to Diverse Glycolipid Antigens

**DOI:** 10.1016/j.immuni.2013.12.004

**Published:** 2014-01-16

**Authors:** Pooja Arora, Andres Baena, Karl O.A. Yu, Neeraj K. Saini, Shalu S. Kharkwal, Michael F. Goldberg, Shajo Kunnath-Velayudhan, Leandro J. Carreño, Manjunatha M. Venkataswamy, John Kim, Eszter Lazar-Molnar, Gregoire Lauvau, Young-tae Chang, Zheng Liu, Robert Bittman, Aymen Al-Shamkhani, Liam R. Cox, Peter J. Jervis, Natacha Veerapen, Gurdyal S. Besra, Steven A. Porcelli

**Affiliations:** 1 Department of Microbiology and Immunology, Albert Einstein College of Medicine, Bronx, NY 10461, USA; 2 Department of Medicine, Albert Einstein College of Medicine, Bronx, NY 10461, USA; 3 Grupo de Inmunología Celular e Inmunogenética GICIG, Departamento de Microbiología y Parasitología, Facultad de Medicina, Universidad de Antioquia UdeA, Calle 70 No.52-21, Medellin 05001000, Colombia; 4 Pediatric Infectious Diseases, Comer Children’s Hospital, University of Chicago, Chicago, IL 60637, USA; 5 Millennium Institute on Immunology and Immunotherapy, Facultad de Medicina, Universidad de Chile, Santiago 8380453, Chile; 6 National Institute of Mental Health and Neuroscience, Bangalore, Karnataka 560029, India; 7 Department of Chemistry and Medicinal Chemistry Programme, National University of Singapore, and Singapore Bioimaging Consortium, Agency for Science, Technology and Research (A^∗^STAR), Biopolis 117543, Singapore; 8 Department of Chemistry and Biochemistry, Queens College of CUNY, Flushing, NY 11367, USA; 9 Faculty of Medicine, Cancer Sciences Academic Unit, University of Southampton, Southampton SO16 6YD, UK; 10 School of Chemistry, University of Birmingham, Edgbaston, Birmingham B15 2TT, UK; 11 School of Biosciences, University of Birmingham, Edgbaston, Birmingham B15 2TT, UK

## Abstract

Many hematopoietic cell types express CD1d and are capable of presenting glycolipid antigens to invariant natural killer T cells (iNKT cells). However, the question of which cells are the principal presenters of glycolipid antigens in vivo remains controversial, and it has been suggested that this might vary depending on the structure of a particular glycolipid antigen. Here we have shown that a single type of cell, the CD8α^+^ DEC-205^+^ dendritic cell, was mainly responsible for capturing and presenting a variety of different glycolipid antigens, including multiple forms of α-galactosylceramide that stimulate widely divergent cytokine responses. After glycolipid presentation, these dendritic cells rapidly altered their expression of various costimulatory and coinhibitory molecules in a manner that was dependent on the structure of the antigen. These findings show flexibility in the outcome of two-way communication between CD8α^+^ dendritic cells and iNKT cells, providing a mechanism for biasing toward either proinflammatory or anti-inflammatory responses.

## Highlights

• Complexes of antigenic glycolipids bound to CD1d have been visualized in situ
• A single DC subset predominates in presentation of a variety of glycolipids
• Antigen presentation to iNKT cells rapidly alters accessory molecules on APCs
• Reciprocal induction of CD70 and PD-L2 controls cytokine bias of iNKT cell responses

## Graphical Abstract

**Figure:**
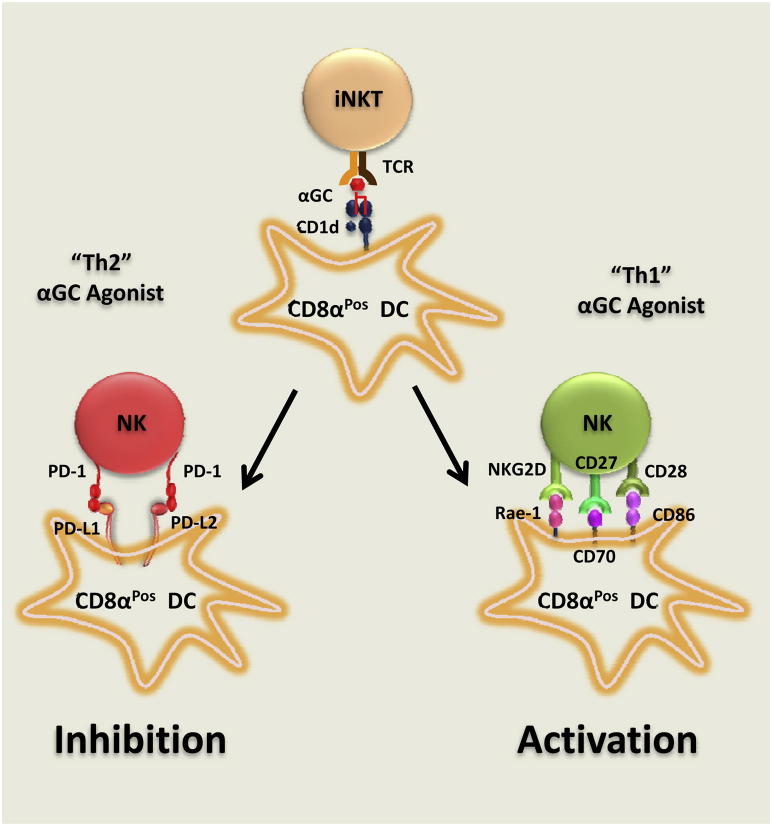

## Introduction

Natural killer T cells with invariant T cell receptor α chains (iNKT cells) are a conserved population that recognizes glycolipid antigens bound to CD1d, a lipid antigen-presenting molecule with structural similarities to major histocompatibility complex (MHC) class I proteins (Brennan et al., 2013, Rossjohn et al., 2012). Studies of the prototypical glycolipid antigen of iNKT cells, an α-galactosylceramide (αGC) known as KRN7000, show the potential for iNKT cells to activate a range of immune effector functions in vivo. This occurs both through direct stimulation of iNKT cell functions and by transactivation of other effectors, most notably NK cells and dendritic cells (Brennan et al., 2013, Carnaud et al., 1999, Taraban et al., 2008). Multiple studies show that this transactivation is influenced by the precise structure of glycolipid antigens, which has enabled manipulation of immune responses with structural analogs of αGC (Venkataswamy and Porcelli, 2010). For example, derivatives of αGC containing truncated or unsaturated N-acyl chains induce responses in which cytokines typically associated with T helper-2 (Th2) cells predominate, and transactivation of NK cells is limited (Yu et al., 2005). On the other hand, replacing the O-glycosidic linkage of αGC with a nonhydrolyzable carbon linker gives a C-glycoside variant that induces cytokine responses biased toward cytokines characteristic of T helper 1 (Th1) cells, along with enhanced transactivation of NK cells and their secretion of interferon-γ (IFN-γ) (Schmieg et al., 2003).

Several models have been put forth to explain how variations in the structure of glycolipid antigens lead to different outcomes of iNKT cell activation. Surprisingly, the induction of Th1 cell- versus Th2 cell-associated cytokines and the extent of NK cell transactivation do not correlate consistently with the potency of different αGC analogs, or with the affinity with which they interact with the T cell receptors (TCRs) of iNKT cells (Im et al., 2009, Sullivan et al., 2010, Tyznik et al., 2011, Wu et al., 2011). A unifying feature of αGC analogs that induce predominantly Th2 cell-associated cytokines is that they are more polar than KRN7000 and can load directly onto CD1d molecules on the cell surface (Im et al., 2009, Tyznik et al., 2011). In contrast, glycolipids that induce responses that are biased toward Th1 cell cytokines are more hydrophobic and require intracellular loading onto CD1d for presentation (Arora et al., 2011, Im et al., 2009). Because most cells of hematopoietic origin express CD1d (Brossay et al., 1997), it has been proposed that selective presentation by different cell types could account for variation in functional outcomes with different glycolipid antigens (Bezbradica et al., 2005, Yu et al., 2005). This possibility was supported by a recent study using lineage-specific conditional deletion of *Cd1d* gene expression, which identified presentation by different types of antigen-presenting cells (APCs) as a major factor underlying the cytokine biasing properties of different αGC variants (Bai et al., 2012). However, other studies yielded different conclusions, identifying pharmacokinetic properties of the glycolipids or localization of CD1d molecules containing bound glycolipids to different membrane microdomains as major determinants of cytokine skewing in the response to iNKT cell activation (Im et al., 2009, Sullivan et al., 2010).

In the current study, we reassessed the presentation of various forms of αGC in vivo to identify the predominant APCs involved in presentation of diverse glycolipid antigens. By visualizing glycolipid antigen presentation directly with monoclonal antibodies specific for complexes of αGC bound to CD1d, we showed that the CD8α^+^DEC-205^+^ subset of dendritic cells was the major APC in the spleen for a range of αGC analogs, irrespective of their chemical structures and cytokine biasing activities. Interaction of CD8α^+^ dendritic cells (DCs) with iNKT cells during presentation of Th1 cell-biasing versus Th2 cell-biasing glycolipid antigens led to markedly different changes in expression of costimulatory and coinhibitory molecules on these cells, including a reciprocal regulation of CD70 and PD-L2 that was linked to enhancing or suppressing IFN-γ production by transactivated NK cells. Our findings suggest that, rather than presentation by alternate types of APCs, the rapid change in cell surface molecule expression by CD8α^+^ DCs in response to different chemical forms of αGC is the principal mechanism regulating bystander cell transactivation and proinflammatory versus anti-inflammatory outcomes following iNKT cell activation.

## Results

### Glycolipid Uptake and Presentation by Candidate APCs

**Figure 1:**
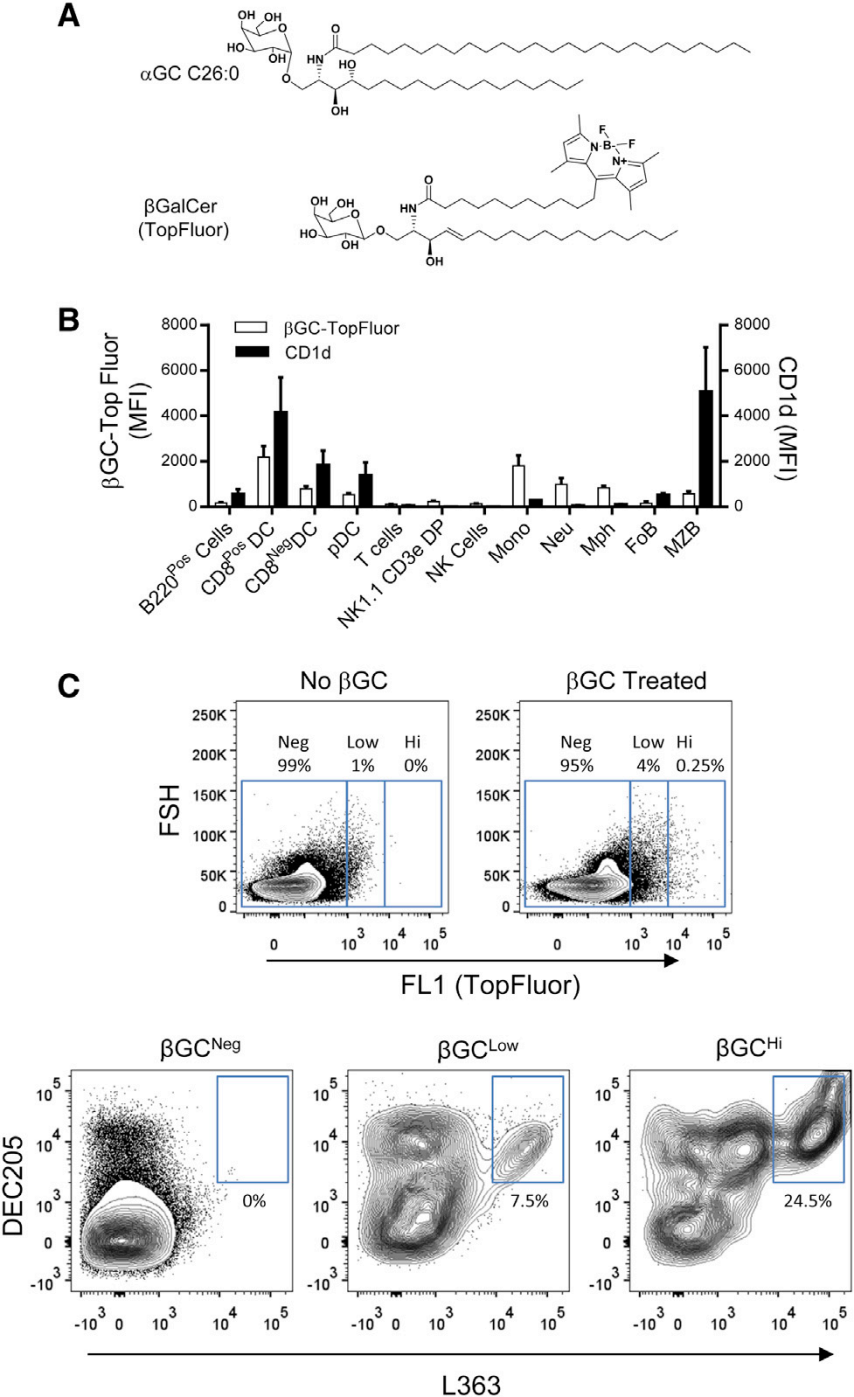
Identification of Cells Mediating Glycolipid Uptake and Presentation in the Spleen (A) Structures of αGC C26:0 and a fluorescently tagged form of βGC. (B) Mice were injected with a mixture of βGC-TopFluor and αGC C26:0 (2 nM of each, i.p.) and spleens were harvested 16 hr later. After staining with mAbs specific for leukocyte markers, flow cytometry was used to gate on each of the indicated cell types and MFI for βGC-TopFluor was determined as a measure of glycolipid uptake (open bars). Staining for total cell surface CD1d expression is also shown (filled bars). Means ± 1 SD are shown for groups of four animals. (C) Plots at top show signal for βGC-TopFluor (FL1) in total splenocytes of representative mice injected i.p. with saline (left) or with 2 nanomoles of βGC-TopFluor (right). Regions were defined as negative, low, or high for βGC-TopFluor staining. Plots below show the βGC-TopFluor negative (left), low (center), and high (right) fractions gated separately and analyzed for DEC-205 expression and αGC C26:0 presentation with the L363 mAb. Numbers indicate frequency of cells in the outlined regions. Representative results of three independent experiments are shown. See also Figure S1.

To identify the cells capable of taking up and potentially presenting glycolipid antigens, we used a fluorescent derivative of β-galactosylceramide (βGC-TopFluor), which has a general overall structural similarity to αGC (Figure 1A) (Boldyrev et al., 2007). After coinjection intraperitoneally (i.p.) of mice with βGC-TopFluor and the CD1d-presented iNKT cell agonist αGC C26:0, splenocytes were harvested and stained with monoclonal antibodies (mAbs) specific for cell surface markers to allow selective gating on each of the major leukocyte subsets. The cells were also stained with a mAb specific for CD1d, or with mAb L363 that selectively recognizes complexes formed by the binding of αGC to CD1d (Yu et al., 2007, Yu et al., 2011). Phagocytic cells including macrophages, DCs, monocytes, and neutrophils were all efficient at taking up the βGC probe, while only minimal uptake was seen with most lymphoid subsets regardless of their CD1d expression (Figure 1B; Figure S1). Gating on the relatively small fraction of highly βGC^+^ cells revealed that ∼25% of these also stained with L363, indicating αGC presentation (Figure 1C). In contrast, a smaller fraction (∼7.5%) of weakly βGC^+^ cells showed L363 binding, and no significant L363 binding was detected among βGC^−^ cells. Further phenotypic analysis indicated that nearly all L363-positive cells expressed the mannose receptor DEC-205 and CD11c, identifying them as a subpopulation of DCs (Figure 1C; data not shown).

**Figure 2:**
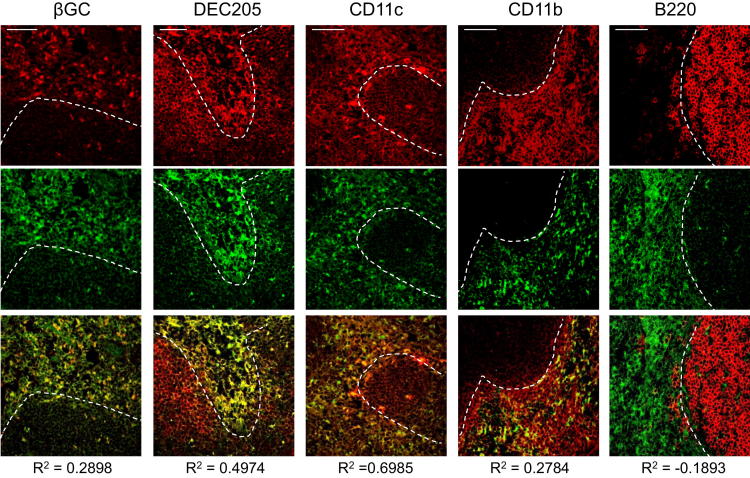
Direct Visualization of Glycolipid Uptake and Presentation in the Spleen Confocal micrographs of splenic sections from αGC C26:0-treated mice (12 hr after i.p. injection) showing showing uptake of βGC-TopFluor or immunofluorescent staining for various phenotypic markers in red (top row) and mAb L363 in green (middle row). Bottom row shows merge of red and green images above each panel. Pearson correlation coefficients (R^2^) for colocalization of red and green signals are shown below merged images. Dashed white lines outline the marginal zone separating T cell rich areas from the B cell rich follicles. Images were acquired using a 40× objective with digital zoom of approximately 2- to 3-fold. Scale bars at the top left of micrographs represent 50 μm. See also Figure S2.

Direct visualization of glycolipid uptake and presentation was achieved with confocal microscopy of splenic cryosections. This showed that L363 staining colocalized partially with βGC-TopFluor, consistent with presentation of αGC by a subset of glycolipid engulfing cells (Figure 2). Interestingly, although CD1d colocalized with both B220^+^ B cells and CD11c^+^ DCs (Figure S2), staining with L363 was largely excluded from B cell areas. Instead, the majority of L363 staining was in the marginal zones and T cell areas of the splenic white pulp, where it colocalized with staining for CD11c and DEC-205. Taken together, these findings indicated that presentation of αGC C26:0 in the spleen following systemic injection was restricted mainly to a population of DEC-205^+^ DCs that avidly take up exogenous glycolipids.

### Identification of In Vivo APCs for αGC Analogs

**Figure 3:**
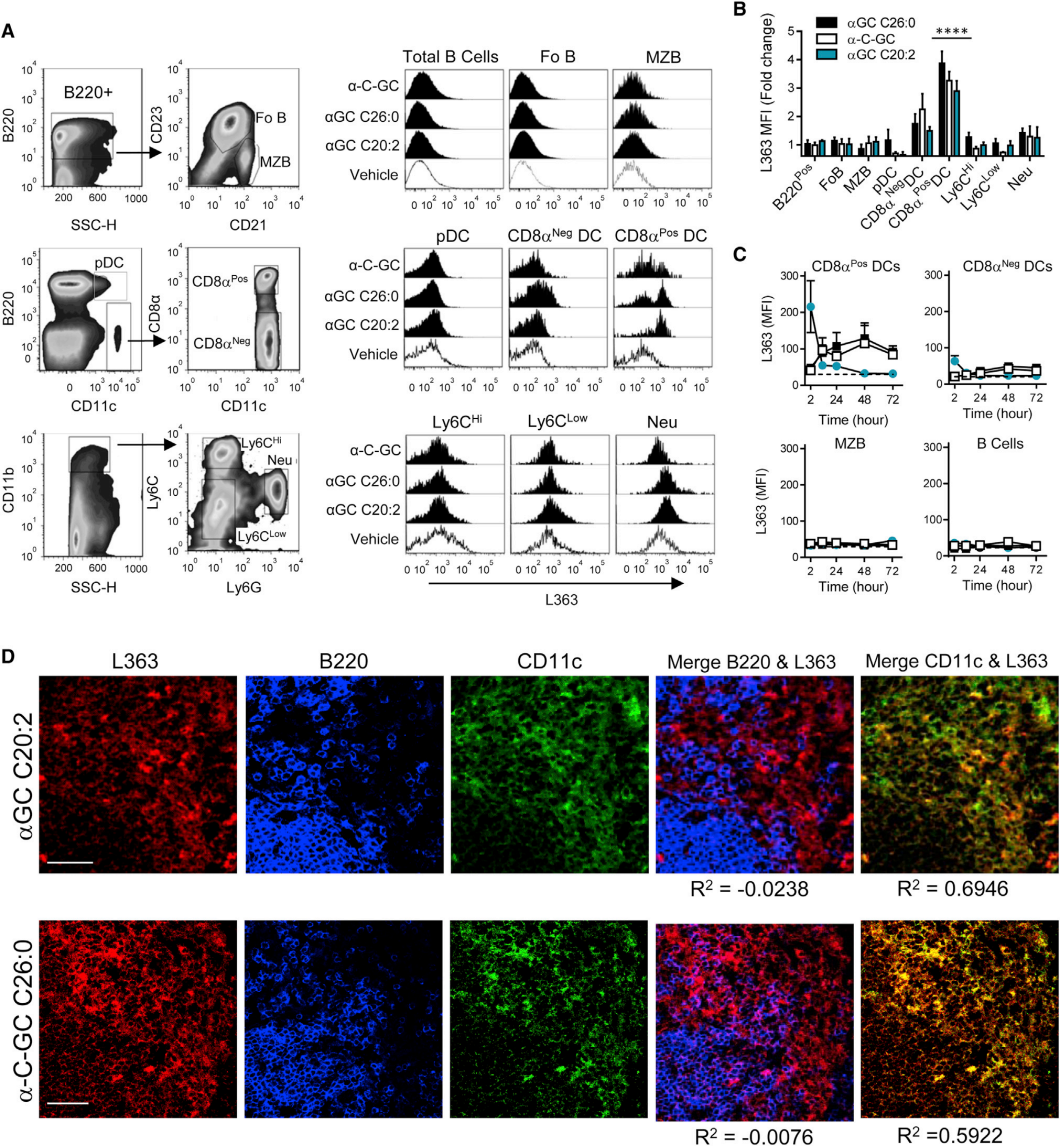
In Vivo Presenting Cells for Functionally Distinct αGC Analogs (A) Flow cytometry of splenic subsets to analyze L363 staining following glycolipid antigen injection. Multiparameter staining for cell-type-specific markers and gating strategy for each population is illustrated by plots on the left. Histograms show the extent of glycolipid presentation based on L363 staining in each cell type from spleens of mice injected previously with αGC analogs that induce different cytokine profiles (filled histograms) or vehicle (open histograms). L363 staining is shown for time points giving maximal signals for each glycolipid (24, 48, and 2 hr after injection for αGC C26:0, α-C-GC, and αGC C20:2, respectively). Fo B, follicular B cells; MZB, marginal zone B cells; pDC, plasmacytoid DCs; Neu, neutrophils. (B) Fold increase in L363 (MFI) over vehicle-injected control mice at the time of peak L363 staining for each of the αGC glycolipids. Data shown are means ± SD, ^∗∗∗∗^p < 0.0001 for comparison with vehicle control (ANOVA with Holm-Sidak test). (C) In vivo kinetics of αGC presentation by B cell and DC subsets based on MFI of L363 staining of splenocytes from animals treated as in (A). Symbols indicate αGC C26:0 (solid squares), α-C-GC (open squares), or αGC C20:2 (solid circles). Dashed line indicates L363 staining of cells from animals receiving vehicle control. Data shown are means ± SEM. (D) Splenic cryosections stained for L363 (red), B220 (blue), and CD11c (green) from mice injected previously with the αGC C20:2 (2 hr after injection) or α-C-GC C26:0 (24 hr after injection). For merged images, R^2^ values are provided as an index of colocalization of the two signals. Scale bars indicate 50 μm. Representative results are shown from at least three independent experiments with analysis of four animals per group in each experiment. See also Figure S3.

We focused on presentation of three well-characterized iNKT cell glycolipid antigens, αGC C26:0, αGC C20:2, and α-C-GC (C-glycoside), which are representative of three different types of cytokine-biasing analogs. Whereas αGC C26:0 induces a mixed response with both Th1 cell- and Th2 cell-associated cytokines, αGC C20:2 stimulates a Th2 cell cytokine pattern and α-C-GC gives a Th1 cell-biased pattern (Figures S3A and S3B). By using flow cytometry, we examined the in vivo presentation of these αGC analogs by different splenic subsets. CD11c^Hi^ DCs showed strong acquisition of L363 staining, which was much higher in CD8α^+^ compared to the CD8α^−^ DCs (Figures 3A and 3B). In contrast, L363 staining was not detected on B cells following injection of any of the glycolipid antigens, including the marginal zone B cells, which showed extremely high CD1d expression. Similarly, presentation was not detected on B220^+^ CD11c^Lo^ plasmacytoid DCs, Ly6C^Hi^ and Ly6C^Lo^ monocytes, or Ly6G^+^ neutrophils. Thus, based on direct detection of glycolipid antigens in complex with CD1d on the cell surface, CD8α^+^ DCs appeared to be the main APC for all three forms of αGC, regardless of their cytokine-biasing properties.

Previous in vitro studies have shown that the kinetics of glycolipid uptake and presentation can vary widely for different αGC analogs, and that this correlates with the type of responses generated (Im et al., 2009, Sullivan et al., 2010). To examine this correlation in vivo, the kinetics of glycolipid presentation by CD8α^+^ and CD8α^−^ DCs, follicular B cells and marginal zone B cells were monitored with the L363 mAb (Figure 3C). This confirmed the rapid presentation of αGC C20:2 by CD8α^+^ DCs and to a lower extent by CD8α^−^ DCs. This presentation was maximal in the spleen by 2 hr after αGC C20:2 injection and then declined rapidly. Both αGC C26:0 and α-C-GC analogs showed slower kinetics of presentation, peaking at 12 to 24 hr and remaining elevated at the latest time points examined (Figure 3C; Figure S3C). In contrast to DCs, no L363 staining was observed on B cells at any time point.

In situ visualization of CD1d molecules containing bound αGC C20:2 or α-C-GC in splenic cryosections by using L363 showed that these failed to colocalize with B220, but strongly colocalized with CD11c (Figure 3D). We also used mice expressing a fusion protein of green fluorescent protein linked to the chemokine receptor CXCR3 (CX3CR1-GFP) to clearly visualize monocytes and macrophages (Swirski et al., 2009), revealing a lack of correlation between GFP-positive and L363-positive cells (Figures S3E and S3F). These findings reinforced our conclusion that CD8α^+^ DCs were the predominant APCs for a range of structurally and functionally distinct glycolipid antigens irrespective of their Th1 cell or Th2 cell cytokine-biased responses. We also found that complexes of CD1d and αGC containing different αGC analogs, formed in vivo on CD8α^+^ DCs, showed different sensitivity to detergent extraction from the plasma membrane (Figure S3D). This finding extends our earlier in vitro observations that correlate the amount of plasma membrane lipid raft localization of CD1d molecules containing bound αGC glycolipids with functional biasing of iNKT cell responses (Arora et al., 2011).

### Requirement for CD8α^+^ DCs for Efficient Glycolipid Presentation

**Figure 4:**
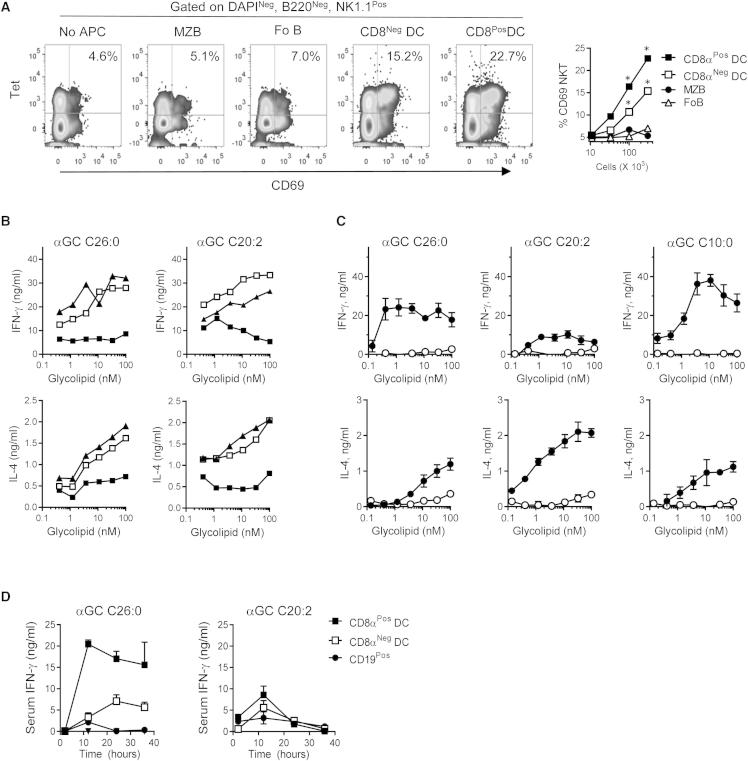
Unique Role of CD8α^+^ DCs in Glycolipid Antigen Presentation (A) Induction of CD69 on iNKT cells showed predominant APC function for CD8α^+^ DCs. Flow cytometry sorting was used to purify MZB, FoB, CD8α^+^, and CD8α^−^ DCs from αGC C20:2 treated mice at 2 hr after glycolipid administration. CD19 and CD11c depleted splenocytes were used as responder cells. Plots show staining with αGC loaded CD1d tetramers and anti-CD69 after 18 hr of coculture for cultures with 3 × 10^5^ APCs. The graph summarizes results with various numbers of APCs. ^∗^p < 0.05. (B) Splenocytes were subjected to immunomagnetic depletion of either CD19 (white squares) or CD11c (black squares) positive cells, or mock depleted (black triangles), and then cultured with various concentrations of αGC C26:0 (left) or αGC C20:2 (right). IFN-γ and IL-4 were quantitated in culture supernatants by ELISA after 16 hr. p < 0.0001 for CD11c^+^ versus CD19^+^ depleted; NS for CD19^+^ versus mock depleted. (C) Purified CD19^+^ (white symbols) and CD11c^+^ (filled symbols) splenocytes were pulsed with different concentrations of three different αGC analogs. After extensive washing, purified NK1.1^+^ spleen cells (enriched ∼20 fold for NK and iNKT cells) were added. Supernatants were collected after 18 hr, and IFN-γ and IL-4 were quantitated by ELISA. p < 0.001 for CD11c^+^ versus CD19^+^ APCs for all glycolipids tested. (D) Purified CD19^+^ (filled circles), CD8α^+^ DCs (Filled squares), and CD8α^−^ DCs (white squares) were pulsed with 100 nM of αGC analogs for 1 hr. Following extensive washing, 1 × 10^6^ of these ex vivo glycolipid pulsed cells were injected i.v., and blood samples were collected at various time points for measurement of serum IFN-γ. Differences were significant for CD8α^+^ and CD8α^−^ DCs compared to CD19^+^ cells (p < 0.001 with αGC C26:0 and p < 0.01 with αGC C20:2). All graphs show means ± SD for triplicate samples, and statistical analyses used two-way ANOVA with Holm-Sidak multiple comparison test. Representative results of two (B and C) or three (A and D) independent experiments with sample size of four mice per group are shown. See also Figure S4.

To further assess the efficiency of αGC presentation by various cell types, we carried out functional assays of the ability of various populations of APCs purified from the spleens of αGC C20:2 injected mice to stimulate normal iNKT cells in culture. By using CD69 upregulation as a sensitive indicator of iNKT cell activation, we observed the strongest activation with purified CD8α^+^ DCs. A lower level of CD69 induction was seen with CD8α^−^ DCs, and little or none by using purified B cell subsets as APCs (Figure 4A). These findings closely recapitulated the pattern of presentation observed with L363 mAb staining (Figure 3B). Very similar results were obtained with other αGC analogs with different cytokine biasing properties with this experimental approach when assessing either upregulation of iNKT cell activation markers (CD69 or CD25) or cytokine production (Figures S4A–S4D).

Additional in vitro studies demonstrated the potent APC function of DCs for αGC C26:0 and other functionally distinct αGC analogs, while also showing that B cells lacked this ability. In one set of experiments, we specifically depleted DCs (CD11c^+^ cells) and B cells (CD19^+^ cells) from splenocyte suspensions and then evaluated their ability to stimulate iNKT cell responses in vitro to αGC C26:0 or αGC C20:2. Compared to B cell depletion, the IFN-γ and interleukin-4 (IL-4) responses to both glycolipids were markedly reduced with DC depletion (Figure 4B). Similar studies using depletion of CD11b^+^ cells, which include monocytes and a majority of macrophages, showed little or no reduction in responses (Figure S4E). To compare the presentation by DCs and B cells directly, these populations were isolated from naive spleens, pulsed in vitro with either αGC C26:0 or αGC C20:2, or with another analog that induces a Th2 cell cytokine bias (αGC C10:0), and then cultured with NK1.1^+^ splenic cells (i.e., iNKT and NK cells) (Figure 4C). With all three glycolipids, there was markedly higher stimulation of IFN-γ and IL-4 secretion with CD11c^+^ DCs as APCs compared to CD19^+^ B cells. Experiments carried out in vivo with purified APC populations treated ex vivo with glycolipid antigens showed the same general pattern (Figure 4D). In this case, we injected mice with identical numbers of purified CD19^+^ B cells, CD8α^−^ DCs, or CD8α^+^ DCs after pulsing them with αGC C26:0 or αGC C20:2. Analysis of serum cytokine secretion showed that CD8α^+^ DCs were the most potent inducers of IFN-γ for both glycolipid antigens. Taken together, these experiments indicated that CD8α^+^ DCs are the predominant APCs, even for glycolipids that induce a strong bias toward production of Th2 cell-associated cytokines.

**Figure 5:**
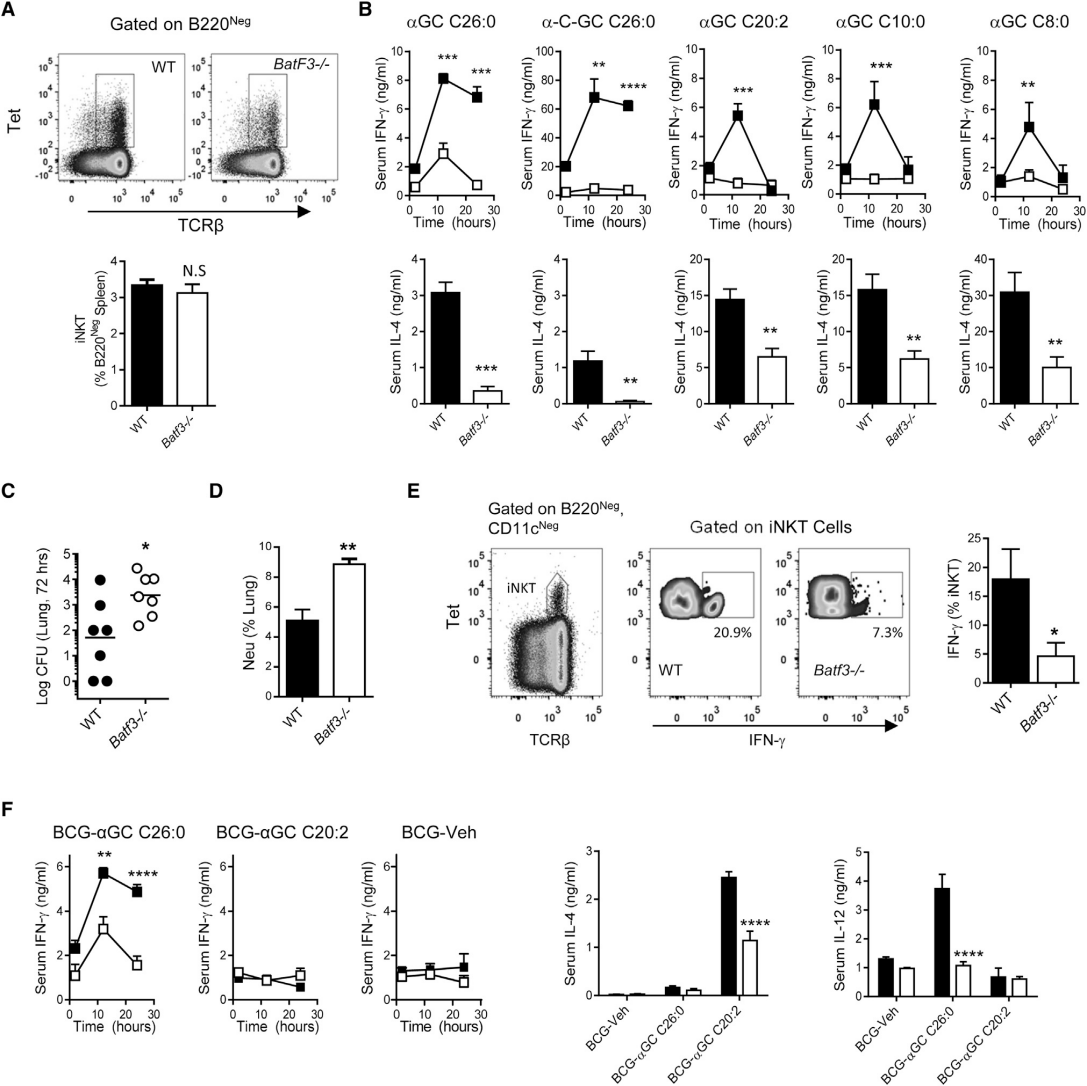
Requirement for CD8α^+^ DCs for Presentation of Synthetic and Natural Glycolipid Antigens (A) Frequency of iNKT cells in *Batf3*^−/−^ and WT naive mice. Representative dot plots show viable B220^−^ cells gated for TCRβ and αGC-loaded CD1d tetramers. Bar graph shows mean ± SD for frequencies of iNKT cells in groups of four mice (NS, not significant; Student’s t test). (B) Comparison of WT and *Batf3*^−/−^ mice for serum cytokine responses to five synthetic glycolipid antigens. All mice were injected i.p. with glycolipids (2 nM for αGC C26:0, α-C-GC, αGC C20:2; 10 nM for αGC C10:0, αGC C8:0). Serum was collected at the time points indicated for measurement of IFN-γ and IL-4 (values for IL-4 are shown only for 2 hr after glycolipid injection). ^∗∗^p < 0.01, ^∗∗∗^p < 0.001, ^∗∗∗∗^p < 0.0001 (two-way ANOVA with Holm-Sidak correction). (C) WT and *Batf3*^−/−^ mice on the 129/SJ background were infected intranasally with 1 × 10^6^*Streptococcus pneumoniae URF918*. Bacterial counts in the lungs on day 2 are shown. ^∗^p < 0.05 (Student’s t test). (D) Neutrophil infiltration in the lungs as determined by flow cytometry. Mice were sacrificed 2 days after intranasal *S. pneumoniae* infection, and the frequency of Ly6G^+^ CD11b^+^ cells as a percentage of total cells in lung suspensions was determined by flow cytometry. ^∗∗^p < 0.01 (Student’s t test). (E) Analysis by flow cytometry of production of IFN-γ by splenic iNKT cells 6 hr after *S. pneumonia* infection. ^∗^p < 0.05 (Student’s t test). (F) Comparison of WT and *Batf3*^−/−^ mice for serum cytokine responses to BCG modified by incorporation of synthetic glycolipid antigens. Mice were injected i.p. with 5 × 10^6^ bacteria and serum cytokines were analyzed. Representative results of three independent experiments with four animals per group per experiment are shown. For the CFU counting experiment seven mice per group per experiment were used. All data are means ± SEM. ^∗∗^p < 0.01 and ^∗∗∗∗^p < 0.0001 (two way ANOVA, Holm-Sidak test). See also Figure S5.

We next sought to investigate the extent to which the CD8α^+^ DCs were required for glycolipid antigen presentation in vivo by taking advantage of mice with deletion of the gene encoding the transcription factor Batf3 (*Batf3*^−/−^ mice). These mice lack CD8α^+^ DCs and have few if any other immunological defects (Hildner et al., 2008). Assessment of iNKT cell frequencies in *Batf3*^−/−^mice showed no evidence of defects in iNKT selection and development (Figure 5A), and comparison of in vitro responses of WT and *Batf3*^−/−^splenocytes to stimulation by αGC-pulsed WT BMDCs indicated that functionality of iNKT cells was not compromised in *Batf3*^−/−^ mice (Figure S5A). However, synthetic αGC analogs induced diminished cytokine responses when injected into *Batf3*^−/−^ mice regardless of the type of αGC used (Figure 5B), indicating a requirement for CD8α^+^ DCs for efficient presentation. The weak IFN-γ responses observed in WT mice to β-glucosylceramide, a candidate self-glycolipid ligand of iNKT cells (Brennan et al., 2013), were also reduced in *Batf3*^−/−^mice (Figure S5B).

The Gram-positive pathogenic bacterium *Streptococcus pneumoniae* is known to contain an α-glycosyl diacyl glycerol glycolipid antigen that is presented by CD1d to iNKT cells, and efficient clearance of this infection in mice requires iNKT cells (Kinjo et al., 2011). To investigate whether microbial glycolipid antigen presentation also required CD8α^+^ DCs in the setting of infection, we compared the lung burdens of *Batf3*^−/−^and WT mice after intranasal infection with *S. pneumoniae*. The *Batf3*^−/−^mice displayed higher bacterial burdens in their lungs (Figure 5C), which was associated with increased neutrophil migration to this site (Figure 5D). This reduction in bacterial clearance correlated with fewer IFN-γ producing iNKT cells in the spleens of infected mice (Figure 5E), indicating that CD8α^+^ DCs were required for iNKT activation by *S. pneumoniae* and for rapid generation of protective immunity to this bacterium.

We have also shown previously that modifying live *Mycobacterium bovis* BCG bacilli by incorporation of αGC enhances its vaccine efficacy against *Mycobacterium tuberculosis* infection (Venkataswamy et al., 2009). By using this approach, we compared serum cytokines in response to i.p. administration of BCG modified by incorporation of either αGC C26:0 or αGC C20:2. In both cases, serum cytokine responses were reduced in *Batf3*^−/−^mice compared to WT animals (Figure 5F), reinforcing the role of CD8α^+^ DCs in efficient glycolipid antigen presentation.

### Modulation of Accessory Molecules on CD8α^+^ DCs

**Figure 6:**
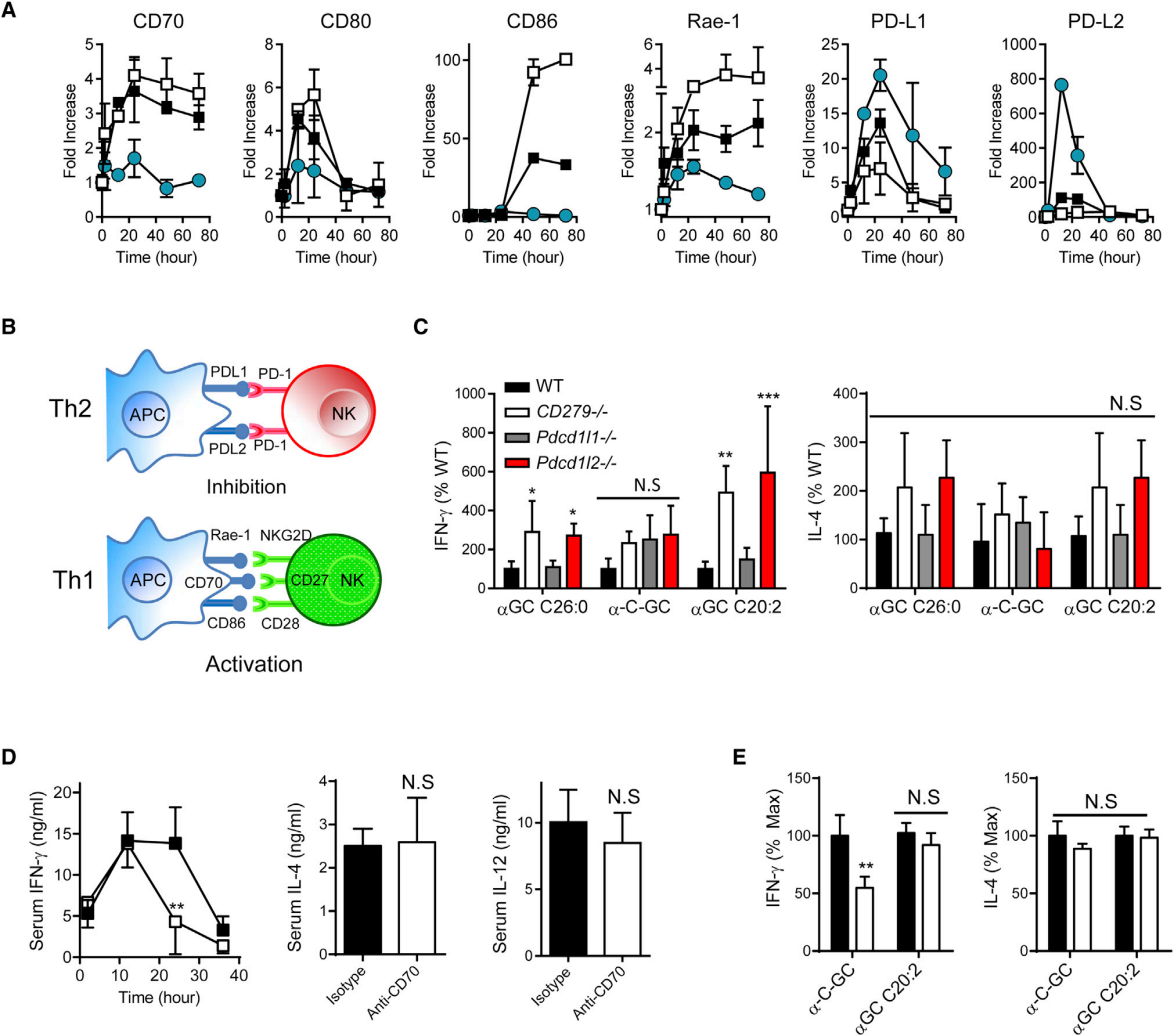
Modulation of Cell Surface Costimulatory and Coinhibitory Molecules on CD8α^+^ DCs following iNKT Cell Antigen Recognition (A) Splenic CD8α^+^ DCs (CD11c^+^ CD8α^+^) were analyzed by flow cytometry for the indicated cell surface molecules at various times following i.p. injection of 2 nM of each αGC analog (filled squares, αGC C26:0; open squares, α-C-GC; blue filled circles, αGC C20:2). Changes in cell surface expression of costimulatory and coinhibitory molecules are shown as the fold increase compared to mice injected with vehicle only. Data shown are means ± SD for groups of three mice. (B) Model for regulation of NK cell transactivation leading to cytokine biasing of iNKT cell-dependent responses through modulation of costimulatory and coinhibitory proteins on APCs. (C) Peak serum cytokines following i.p. injection of αGC glycolipid antigens into WT or mice with genetic ablation of PD-1, PD-L1, or PD-L2 (*Cd279*^−/−^, *Pdcd1lg2*^−/−^, *Pdcd1lg2*^−/−^). The indicated glycolipids (αGC C26:0, α-C-GC, or αGC C20:2) were injected i.p. (2 nM/mouse). IFN-γ was measured at 24 hr (for αGC C26:0 and α-C-GC), or at 12 hr for αGC C20:2. IL-4 was measured at 2 hr after glycolipid injection for all analogs. Data are shown as means ± SD for groups of three mice, calculated as percent of WT response for each αGC analog. (D) Effects of in vivo pretreatment of WT mice with anti-CD70 blocking antibodies (open symbols and bars) compared to isotype matched control antibody (filled symbols and bars). Mice received 200 μg of anti-CD70 or isotype matched control antibody i.p., and 16 hr later were injected with αGC C26:0 (2 nM i.p.). Serum cytokines were measured at the indicated times after glycolipid injection for IFN-γ, and at 2 hr for IL-4 or 8 hr for IL-12. (E) For α-C-GC and αGC C20:2 glycolipid agonists, isotype control (filled bars), and anti-CD70 (open bars) treated groups are compared for serum IL-4 and IFN-γ responses. IFN-γ was measured at 24 hr for α-C-GC and 12 hr for αGC C20:2 agonists, and IL-4 was measured at 2 hr in both cases. Representative results of three independent experiments with four mice per group per experiment are shown. All data are means ± SD. For (C), (D), and (E): ^∗^p < 0.05, ^∗∗^p < 0.01, ^∗∗∗^p < 0.001; NS, not significant (two-way ANOVA with Holm-Sidak correction). See also Figure S6.

The outcomes of cellular interactions during immune responses are controlled to a great extent by costimulatory and coinhibitory proteins, which are inducible on APCs in a regulated manner. This raised the possibility that different analogs of αGC might induce different patterns of costimulatory or coinhibitory proteins on the surface of CD8α^+^ DCs. We therefore analyzed changes in expression of multiple costimulatory or coinhibitory molecules by CD8α^+^ DCs over a 3 day period after injection of mice with either αGC C26:0, C20:2 or C-glycoside. Although some of these molecules (CD40 and CD80) showed a similar upregulation with all three of the glycolipid antigens, others including CD70, Rae-1, and CD86 showed prominent upregulation only with the glycolipids that induced strong Th1 cell-associated cytokine responses. Conversely, the coinhibitory proteins PD-L1 and PD-L2 were upregulated most prominently on DCs presenting αGC C20:2 that induces Th2 cell cytokine-biased responses (Figure 6A; Figure S6A). Modulation of costimulatory and coinhibitory molecules in response to iNKT cell agonists were seen almost exclusively on CD8α^+^ DC, with little change in expression on CD8α^−^ DCs, plasmacytoid DCs or B cells (Figure S6B). All of these alterations appeared to be dependent on iNKT cells because they did not occur in *Tcra-J*^*tm1Tgi*^ mice (commonly referred as Jα18^−/−^) lacking iNKT cells (Figure S6C; additional data not shown). In addition, we transferred purified CD11c^+^ DCs from *Cd1d* ^−/−^ and WT animals into WT hosts and analyzed CD70 expression after glycolipid administration. This showed greater induction of CD70 on transferred WT CD8α^+^ DC compared to transferred *Cd1d* ^−/−^ DCs (Figure S6D), suggesting involvement of a direct CD1d-dependent interaction between iNKT cells and APCs in this process.

The observed patterns of modulation of CD70, Rae-1, PD-L1, and PD-L2 by CD8α^+^ DCs provided a plausible mechanism for controlling the cytokine-biasing activities seen with different structural analogs of αGC. Thus, a model can be proposed in which the reciprocal modulation of costimulatory and coinhibitory molecules on CD8α^+^ DCs leads to differences in the amount of transactivation of other innate effectors such as NK cells (Figure 6B). We tested this by studying the roles of PD-1, PD-L1, PD-L2, and CD70 in modulating cytokine responses stimulated by different αGC analogs. For PD-1 ligands, this was done by measuring serum cytokine responses to injections of αGC analogs in PD-1, PD-L1, and PD-L2 deficient mice. This indicated a major role for PD-L2 but not PD-L1 interactions in modulating iNKT cell dependent cytokine responses to reinforce the bias toward Th2 cell cytokines induced by αGC C20:2 (Figure 6C). Thus, PD-L2 signaling had a major effect on dampening IFN-γ production stimulated by αGC C20:2, whereas this was much less evident for αGC C26:0 and absent for α-C-GC. While the proportion of NK cells expressing IFN-γ increased significantly in mice genetically ablated of PD-1 and PD-L2 molecules in response to αGC C26:0 (Figure S6G), serum IL-12 did not increase relative to WT animals in mice lacking PD-1, PD-L1, or PD-L2 receiving this glycolipid (Figure S6F), suggesting that the mechanism for this did not involve an increase in availability of IL-12. In contrast to the PD-1 signaling effects, a reciprocal role was demonstrated for CD70 in experiments with treatment of mice with anti-CD70 blocking antibodies prior to glycolipid administration. Consistent with the proposed model, blockade of CD70 reduced IFN-γ secretion by αGC C26:0 and α-C-GC, but had no significant effect on either IL-12 or IL-4 production stimulated by these analogs or on the predominantly Th2 cell-associated cytokine responses induced by αGC C20:2 (Figures 6D and 6E; Figure S6E). Overall, our results demonstrate the central role of CD8α^+^ DCs in the presentation of a range of different glycolipid antigens and define a mechanism by which these APCs control the outcome of iNKT cell activation through the modulation of cell surface costimulatory molecules.

## Discussion

CD1d is widely expressed by various hematopoietic cells and the relative importance of different cell types in glycolipid antigen presentation in vivo remains ambiguous. Previous studies have suggested that the type of APC involved in glycolipid antigen responses might vary depending on the structure of the glycolipid (Bai et al., 2012, Bezbradica et al., 2005, Moody et al., 2002), leading to major differences in cytokine responses. Here we have reexamined these issues by using a variety of approaches to track glycolipid uptake and presentation. Our results identified CD8α^+^DEC-205^+^ DCs as the key APCs for a range of structurally different glycolipid antigens and suggested a mechanism for control of iNKT cell responses through the modulation of costimulatory and coinhibitory molecules on these DCs.

Most phagocytic cells showed strong uptake of a fluorescent glycolipid probe, which might reflect either increased rates of bulk extracellular fluid uptake or receptor-mediated endocytosis (Freigang et al., 2012, van den Elzen et al., 2005). Macrophages are known to express CD1d, although relatively weakly, and are likely to interact with iNKT cells. Indeed, recent studies have provided evidence for involvement of macrophages glycolipid capture and initial antigen presentation events in iNKT cell responses (Barral et al., 2010, Barral et al., 2012, Lee et al., 2010). The presenting function of macrophage in those studies, as opposed to the predominance of CD8α^+^ DCs in our experiments, might be due to the use of particulate forms of glycolipid antigen such as intact spirochetes (Lee et al., 2010) and 200 nm silica-based particles (Barral et al., 2010) requiring macrophages for efficient capture and processing. This appears not to be required for the free glycolipid antigens we have studied, nor for the two bacterially delivered antigens we have employed. In addition, studies showing presentation by macrophages have used mainly effects on positioning and migration of iNKT cells to detect activation. These effects might be more sensitive indicators of activation than cytokine production or transactivation of NK cells, which are the main end points in our experiments. Thus, while presentation by macrophages is likely to occur in some contexts, our results identified a dominant role for CD8α^+^ DCs at least for a subset of glycolipid antigens and for certain types of iNKT cell responses. Of note, another recent study arrived at a similar conclusion regarding the predominance of DCs in presentation of αGC and *S. pneumonia* to iNKT cells in vivo (King et al., 2013).

A striking finding of our experiments was that the predominance of CD8α^+^ DCs as APCs was observed with multiple different forms of αGC, including those that induce either a bias toward Th1 cell or Th2 cell-associated cytokine responses. Our results in *Batf3*^−/−^ mice also indicated a predominant and nonredundant role for CD8α^+^ DCs in CD1d-mediated presentation. These results suggest different conclusions than those of a recent study in which *Cd1d1*^flox/flox^ mice were used to ablate expression of CD1d in DCs, B cells, and macrophages to assess the role of these cells in presentation of αGC variants (Bai et al., 2012). Using this approach, it was concluded that forms of αGC that induce a bias toward Th2 cell-associated cytokines can be presented by either DCs or B cells, and that preferential presentation by B cells accounts for the altered cytokine pattern seen with these agonists. Like those investigators, we also observed partial maintenance of IL-4 responses to glycolipids of this type in the absence of CD8^+^ DCs (i.e., in *Batf3*^−/−^mice in our case; see Figure 5B). Overall, the results are consistent with a limited ability of other CD1d-positive cells distinct from DCs to present αGC to iNKT cells. However, in the normal situation with CD8α^+^ DCs present, our data suggest that this alternative APC function of B cells or other types of CD1d expressing cells is active at a relatively low level and might not contribute significantly to iNKT cell responses.

Taken together, our results appear inconsistent with a central role for alternate types of APCs in generating the functionally different outcomes following iNKT cell activation, at least for the selected range of antigens we have studied. Instead, we propose that there is a more subtle mechanism operating at the subcellular level that is mainly responsible for the selective induction of the so-called Th1 cell- and Th2 cell-associated cytokine-biased responses. Whereas efforts to attribute cytokine-biasing effects of glycolipid antigens to variations in DC-derived cytokines have led to conflicting results (Forestier et al., 2007, Miyamoto et al., 2001), we found in a previous study that induction of NK cell transactivation and sustained IFN-γ secretion correlates with the extent to which different αGC analogs are presented by CD1d molecules localized in lipid raft microdomains on the APC plasma membrane (Im et al., 2009, Tyznik et al., 2011). This suggests parallels with MHC class II-restricted T cell responses, which have also been found to be regulated by lipid raft-dependent signaling through peptide-MHC II complexes on DCs (Meyer zum Bueschenfelde et al., 2004). We hypothesize that recognition by iNKT cells of lipid raft localized CD1d molecules containing bound αGC glycolipids induce a different intracellular signaling cascade compared with recognition of non-lipid raft localized CD1d molecules, thus leading to the markedly divergent changes in expression of cell surface and secreted molecules by CD8α^+^ DCs. Consistent with this, we found that remodeling of the costimulatory and coinhibitory molecule profiles on APCs could provide a mechanism for the contrasting responses to different αGC analogs. Although the transactivation of NK cells and other relevant cells is likely to be influenced by multiple factors (Bricard et al., 2010, Brigl et al., 2003, Lind et al., 2009), our data suggest that changes in CD70 expression induced by iNKT cells might transactivate NK cells directly through interaction with CD27. In addition, selective upregulation of Rae-1 and CD86 by glycolipid antigens that stimulate strong Th1 cell-associated cytokines could potentially synergize with activating signals from CD27-CD70 interactions through binding to their cognate receptors NKG2D and CD28, which are both expressed by NK cells and other subpopulations of lymphocytes.

In addition to the effects of iNKT cells on positive regulators of transactivation, we also observed reciprocal effects on PD-1 ligands that are negative regulators of immune responses. Most studies have shown PD-L1 to be the dominant interacting partner for PD-1, and this interaction reduces TCR signaling by initiating a dephosphorylation cascade (Keir et al., 2008). Although previous studies have found evidence for effects of both PD-L1 and PD-L2 on glycolipid-induced iNKT cell responses (Singh et al., 2011), we observed significant effects on such responses in *Pdcd1lg2*^−/−^ but not in *Pdcd1lg1*^−/−^ mice. Taken together, the strong and rapid upregulation of PD-L2 observed on APCs following recognition of Th2 cell-biasing glycolipids such as αGC C20:2, along with increased cytokine responses in *Pdcd1lg2*^−/−^ mice that lack PD-L2 protein, implicated selective upregulation of PD-L2 on APCs as a mechanism to restrain the transactivation of bystander cells. This mechanism is likely to play a role in suppressing NK cell-mediated IFN-γ production, thereby reinforcing the cytokine profile induced by glycolipid antigens that give a Th2 cell cytokine bias.

In summary, our results demonstrate a predominant role for the CD8α^+^ DC subset in presentation of a variety of glycolipid antigens, including examples of those that elicit markedly different patterns of cytokines. Rather than supporting the hypothesis that presentation by different types of APCs is the major mechanism responsible for the divergent responses to various glycolipids, our results suggest that these responses are regulated mainly by a mechanism intrinsic to CD8α^+^ DCs that determines the extent to which NK cells and other leukocytes are rapidly transactivated. This mechanism is controlled by iNKT cells through their recognition of CD1d-presented glycolipids on CD8α^+^ DCs, and involves modulation of the landscape of cell surface costimulatory and coinhibitory molecules expressed by these DCs in a manner that is dependent on the fine structure of glycolipid antigens. Although the details of the signaling processes through which iNKT cells modulate CD8α^+^ DC functions remain to be determined, further studies of this process should facilitate the application of glycolipid activators of iNKT cells as vaccine adjuvants or in other types of immunotherapeutic interventions.

## Experimental Procedures

### Mice

Mice were maintained under specific pathogen-free conditions and used at 6–8 weeks of age. The *CD279*^−/−^, *Pdcd1lg1*^−/−^, and *Pdcd1lg2*^−/−^ mice were provided by Dr. Stanley Nathenson. All other mice were purchased from Jackson Laboratories. For *S. pneumoniae* infection studies, *Batf3*^−/−^ and control WT mice were 129SJ background. All other mice were backcrossed to C57BL/6 for ≥10 generations. Blood samples were collected at 2, 12, and 24 hr after APC transfer or glycolipid administration. For IL-12 analysis, samples were collected at 8 hr after glycolipid administration. All procedures involving animals were approved by the Institutional Animal Care and Use Committee.

### Reagents, Antibodies, and Glycolipids

Chemical reagents were purchased from Sigma-Aldrich unless specified otherwise. The mAb L363, specific for the complex composed of mCD1d with a bound αGC glycolipid, was produced in our laboratory (Yu et al., 2007) and conjugated to Alexa Fluor 647 (Invitrogen). Fluorochrome conjugates of mAbs specific for cell markers and cytokines were purchased from BD Biosciences, eBiosciences, and Invitrogen. The anti-CD70 mAb used for in vivo blocking studies (TAN1-7) is a rat IgG2a that was generated by standard fusion techniques following immunization with soluble recombinant mouse CD70-Fc fusion protein (A.A.-S., unpublished data). Mice received 200 μg i.v. of anti-CD70 or isotype-matched control antibody 16 hr before glycolipid administration. Glycolipids were synthesized and prepared as 20 μM solutions for injection in vehicle (PBS + 0.1% DMSO + 0.05% Tween-20) as described previously (Im et al., 2009, Tyznik et al., 2011). Mice were injected i.p. with 0.1 ml of these solutions for a glycolipid dose of 2 nM. β-glucosylceramide (C18 sphingosine and C24:1 acyl group) was purchased from Avanti. For in vitro assays, glycolipids were dissolved in 100% DMSO at 500 μM, briefly sonicated, and then diluted directly into prewarmed (37°C) culture medium.

### Cell Culture

Tissue culture reagents were purchased from Invitrogen. Cells were cultured in U bottom 96-well plates at a density of 2.5 × 10^5^ cells per well in complete RPMI-1640 media containing 10% FBS. For the cell-depletion assays, splenic cells were cultured with a range of different glycolipid concentrations after mock, CD11c, CD19, or F4/80 depletion. Biotinylated monoclonal antibodies specific for CD11c, F4/80, CD19 or NK1.1, and anti-biotin magnetic beads (Miltenyi) were used for depletion or enrichment experiments. For the in vitro APC potency assays, isolated cells were pulsed with range of glycolipid concentration for 1 hr, washed 5 times with PBS, and cocultured with NK1.1^+^ cells for 16 hr. Supernatants were analyzed for secreted IFN-γ and IL-4 by using capture ELISA as described previously (Im et al., 2009, Tyznik et al., 2011).

### Bacterial Infections

The *Streptococcus pneumoniae* URF918 strain was obtained from Dr. M. Kronenberg. Bacterial cultures were grown in Todd-Hewitt Broth media at 37°C with 5% CO_2_ to mid log phase as described (Kinjo et al., 2011). Mice were inoculated intranasally with 1 × 10^6^ colony forming units of *S. pneumoniae*. Murine lungs and spleens were harvested 48 or 72 hr after infection, and bacterial burdens were estimated by plating the tissue homogenates on blood agar media plates. Incorporation of glycolipids into BCG was performed as described earlier (Venkataswamy et al., 2009) with minor modifications.

### Flow Cytometry

Spleen and lung tissues were minced with a scalpel and digested with Liberase and DNase, and passed through a 70 μm filter to generate single cell suspensions. For intracellular staining, the cells were stained with antibodies to cell surface markers before fixation with 2% paraformaldehyde. Cells were then treated with permeabilization buffer (1% BSA, 0.1% sodium azide, 0.05% glycine, and 0.05% Triton X-100), then blocked with 10% mouse serum and finally stained for 15 min on ice with antibodies to specific cytokines. Samples were analyzed with FACSCalibur, FACSCanto II, or LSR II cytometers and sorted with a FACSAria cell sorter (BD Biosciences).

### Immunofluorescence Microscopy

Fixed and sucrose impregnated tissues were embedded in OTC freezing media for cryosectioning. The frozen sections were permeabilized with 0.5% saponin, blocked with 10% mouse serum, and stained with appropriate antibodies. After washing, the sections were mounted with ProLong® Gold (Invitrogen). Confocal images were acquired with a Leica SP5 AOBS (Leica Microsystems). Image analysis and calculation of Pearson correlation coefficients (R^2^) to assess colocalization of fluorescent signals were done with ImageJ software (Schneider et al., 2012).

### In Vivo APC Transfer Assays

For cell transfers, CD19^+^, CD11c^+^, and CD8^+^ DC isolation reagents (Miltenyi) were used to enrich the cell populations from naive murine splenocytes. For isolation of monocytes and neutrophils, splenocytes were depleted of CD19^+^ cells and sorted after staining for CD11b, Ly6C, and Ly6G on a FACSAria cell sorter. All isolated subsets used for experiments were of >95% purity. After enrichment, cells were pulsed with 100 nM glycolipid in complete RPMI-1640 media for 1 hr, washed 5 times with PBS and counted for live cells with trypan blue exclusion. One million cells were injected intravenously in 0.2 ml volume. For the dendritic cell adoptive transfer experiments, CD11c^+^ cells were purified from WT and *Cd1d*^−/−^ animals by using anti-CD11c magnetic beads (Miltenyi Biotech), labeled with CFSE and injected i.v. an hour before glycolipid administration.

### Statistical Analysis

Student’s t test was used for comparisons involving two groups. One way ANOVA with Dunnet’s correction was used for comparing groups of three or more when there was only one independent variable. Two-way ANOVA with Holm-Sidak correction was used to analyze the effect of glycolipid treatment and BatF3 deletion as two independent factors. Statistical tests were performed with Prism 6 software (GraphPad).
