# Supplementary material for: A Single Subset of Dendritic Cells Controls the Cytokine Bias of Natural Killer T Cell Responses to Diverse Glycolipid Antigens
Source: Immunity. 2014 Jan 16;40(1):105–16. doi: 10.1016/j.immuni.2013.12.004 (PMC3895174; doi:10.1016/j.immuni.2013.12.004)
Supplement: Document S1. Figures S1–S6 [file mmc1.pdf]

Supplemental Information

A single subset of dendritic cells controls the cytokine bias of natural killer T cell responses to diverse glycolipid antigens

Pooja Arora, Andres Baena, Karl O. A. Yu, Neeraj K. Saini, Shalu S. Kharkwal, Michael F. Goldberg, ShajoKunnath-Velayudhan, Leandro J. Carreño, Manjunatha M. Venkataswamy, John Kim, Eszter Lazar-Molnar, GregoireLauvau, Young-tae Chang, Zheng Liu, Robert Bittman, Aymen Al-Shamkhani, Liam R. Cox, Peter J. Jervis, NatachaVeerapen, Gurdyal S. Besra and Steven A. Porcelli

Inventory of Supplemental Information

- Figure S1, related to Figure 1
- Figure S2, related to Figure 2
- Figure S3, related to Figure 3
- Figure S4, related to Figure 4
- Figure S5, related to Figure 5
- Figure S6, related to Figure 6

Figure S1

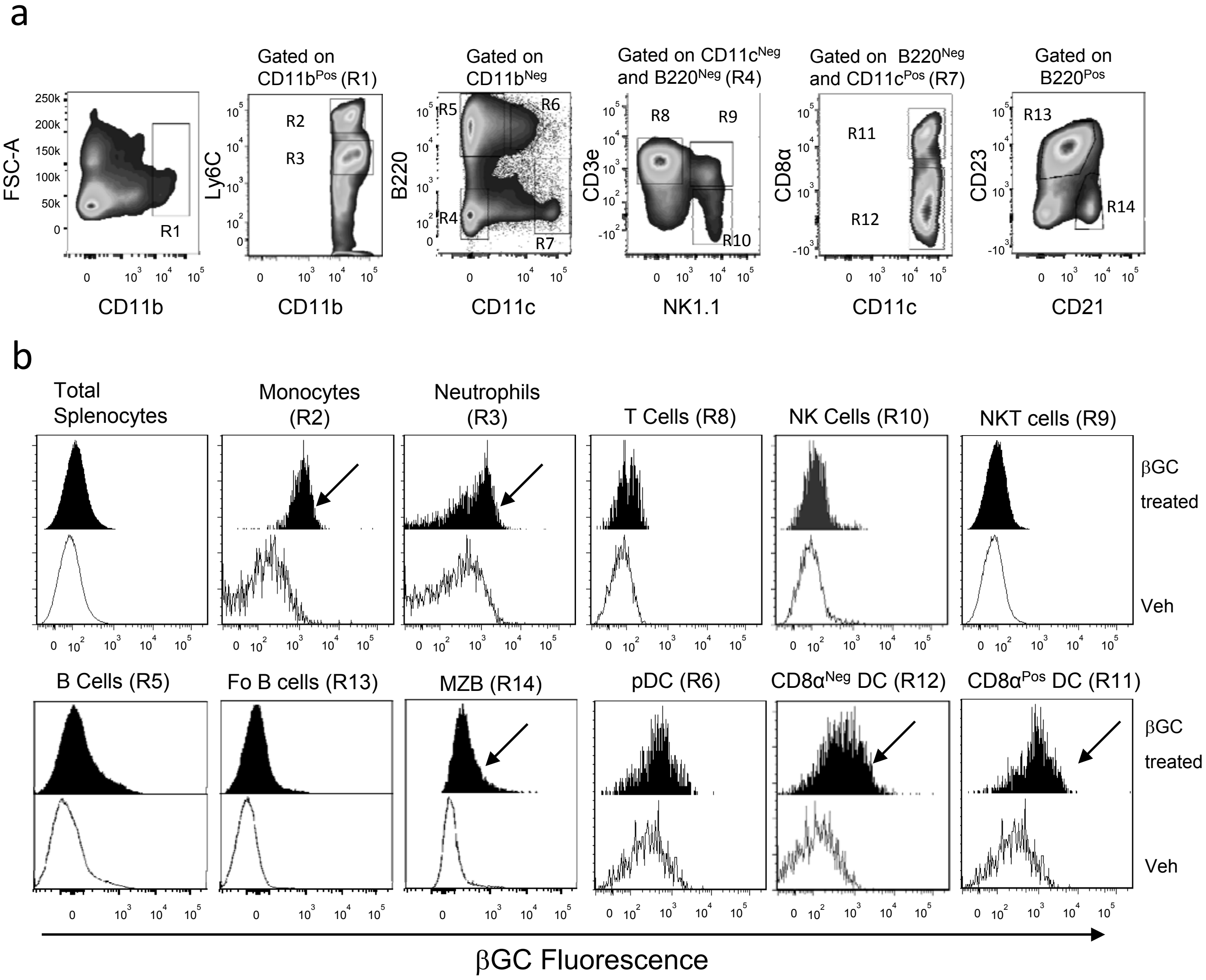

Figure S1, Related to Figure 1: Multiparameter flow cytometry analysis for identification of leukocyte subsets and quantitation of fluorescent glycolipid uptake. **(a)** Gating strategy for identification of various cell types in spleen cell suspensions using 11 color flow cytometry (9 fluorochrome labeled antibodies as shown plus live/dead viability dye and one channel for βGC-TopFluor fluorescent glycolipid probe). **(b)** Filled histograms show signal for βGC-TopFluor for each of the separately gated cell types from spleens of mice injected 16 hr previously with the glycolipid. Open histograms below show the level of background fluorescence in mice injected with vehicle only. The arrows indicate populations that are clearly shifted with respect to the background fluorescence, indicating fluorescent glycolipid uptake. Three mice per group were used in the experiment. Results are representative of three independent experiments.

Figure S2

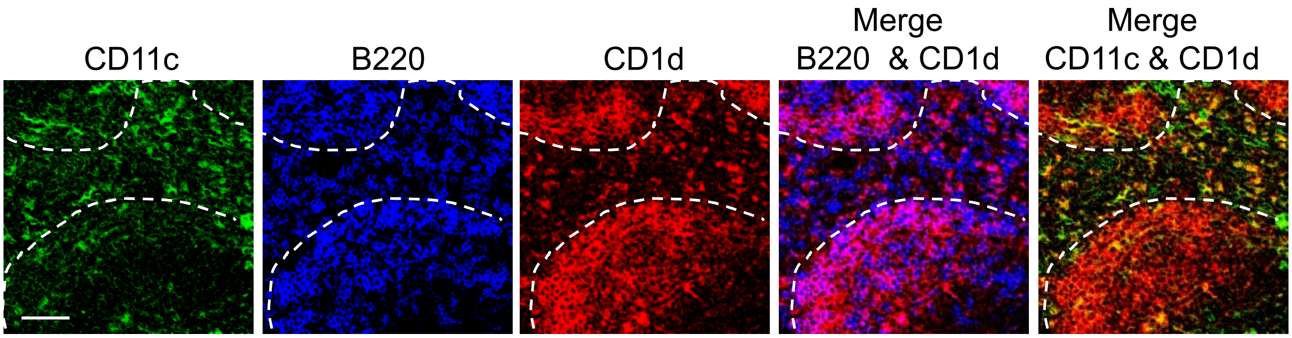

Figure S2, related to Figure 2: Confocal immunofluorescence microscopy of splenic cryosections from normal control mice (green, CD11c; blue, B220; red, CD1d). Dashed lines outline the splenic follicles. High CD1d expression can be seen on B220 positive cells lining the follicle and CD11c positive cells located in the interfollicular zone. The scale bar corresponds to 50 µm.

Figure S3

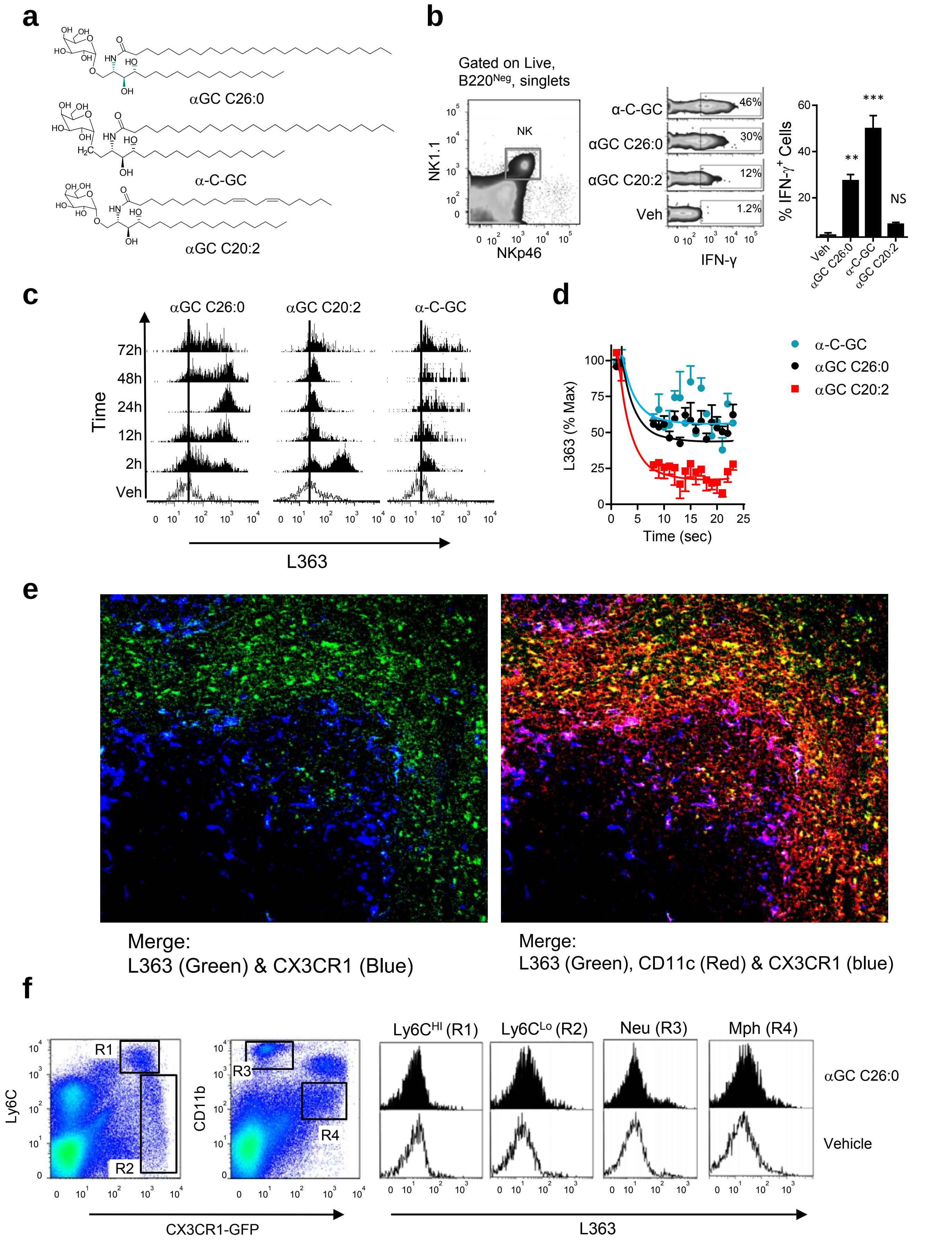
a
αGC C26:0
α-C-GC
αGC C20:2
b
Gated on Live, B220Neg, singlets
NK
NK1.1
NKp46
α-C-GC
αGC C26:0
αGC C20:2
Veh
46%
30%
12%
1.2%
IFN-γ
% IFN-γ+ Cells
**
***
NS
Veh
αGC C26:0
α-C-GC
αGC C20:2
c
αGC C26:0
αGC C20:2
α-C-GC
72h
48h
24h
12h
2h
Veh
Time
L363
d
α-C-GC
αGC C26:0
αGC C20:2
L363 (% Max)
Time (sec)
e
Merge:
L363 (Green) & CX3CR1 (Blue)
Merge:
L363 (Green), CD11c (Red) & CX3CR1 (blue)
f
R1
R2
R3
R4
Ly6C
CD11b
CX3CR1-GFP
Ly6CHI (R1)
Ly6CLo (R2)
Neu (R3)
Mph (R4)
αGC C26:0
Vehicle
L363

Figure S3, related to Figure 3: Cytokine biasing αGC analogues. **(a)** Structures of the αGC analogues used in this study. **(b)** Production of intracellular IFN-γ by NK cells (NK1.1 and NKp46 double positive). Splenocytes were harvested from mice at 12 hr post injection i.p. with αGC analogues and stained for intracellular IFN-γ. $B220^{Pos}$ and dead cells were excluded before gating on the NK1.1 and NKp46 double positive cells (left). Plots (center) show the percentage of IFN-γ positive gated NK cells in response to each of the different glycolipids compared to vehicle injected mice. Mean percentages with standard deviations of IFN-γ positive NK cells for groups of mice (N = 4) injected with each αGC analogue or vehicle only are shown in the graph (right). ** *P < 0.01 and* *** *P < 0.001* (ANOVA, Dunnett post test). **(c)** Kinetics of presentation of αGC analogues by $CD8\alpha^{Pos}$ DCs. Mice were injected with 2 nanomoles of each αGC analogue and spleen cells were harvested at 2, 12, 24, 48 and 72 hr post glycolipid administration. $CD8\alpha^{Pos}$ cells were gated as CD11cPos and $CD8\alpha^{Pos}$ cells, and costained with mAb L363 to complexes of CD1d loaded with αGC analogues on the cell surface. Filled histograms indicate cells from mice injected with the indicated form of αGC, and open histograms show background staining on cells from vehicle-treated control mice. Three mice per group were used for each time point and representative histogram plots are shown for individual time points. **(d)** $CD8\alpha^{Pos}$ DCs were purified by fluorescence activated cell sorting from mice treated with αGC C26:0, α-C-GC and αGC C20:2 αGC analogues at 12, 16 and 2 hrs post glycolipid administration. Three mice per group were used and tissues were pooled before sorting. Sorted cells were monitored for mAb L363 staining before (time = 0 sec) and after exposure to 0.05% Tx-100. Decrease in L363 staining after detergent additions is plotted as percent of staining obtained before detergent addition with each individual glycolipid analogue. Samples were analyzed in triplicate, and means with standard deviations are plotted. **(e)** Phenotypic characterization of *in vivo* glycolipid antigen presenting cells in CX3CR1-GFP mice. CX3CR1-GFP mice (BALB/c background) were treated with αGC C26:0 (2 nanomoles, i.p.). Cryosections of the spleen were prepared at 12 hr post glycolipid treatment. These mice have large, strongly GFP positive cells lining the follicles which belong to the macrophage lineage, and also have $CD11c^{Pos}$ cells that are weakly GFP positive. As shown in the merged image on the left, very little colocalization is seen for L363 staining in these mice with the GFP positive large cells (shown in blue). Merged image on the right shows strong colocalization of L363 staining with CD11c. **(f)** Flow cytometric analysis of CX3CR1-GFP mice. Gating strategy is shown for the separation of monocytes into GFP positive Ly6C low and high populations. Also, the neutrophils (Neu, GFP^{Neg}) and macrophages (Mph, intermediate for GFP and CD11b) are easily identified in these mice. Histograms show minimal or no increase for L363 staining in these myeloid cell populations from glycolipid treated mice (black) compared to vehicle treated mice (white). Three mice per group were used and data are representative of two independent experiments.

Figure S4

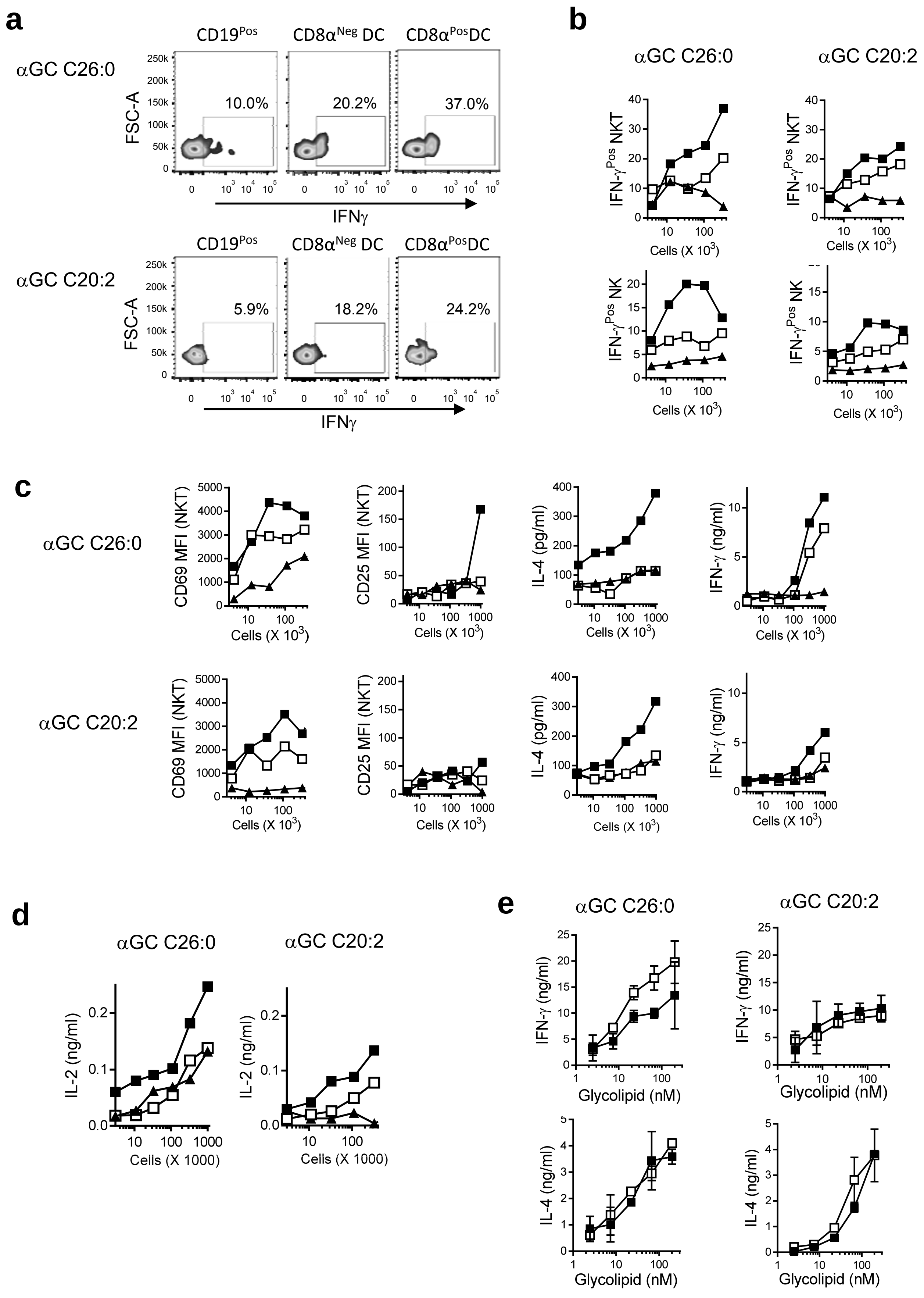
a
αGC C26:0
CD19Pos
CD8αNeg DC
CD8αPosDC
FSC-A
10.0%
20.2%
37.0%
IFNγ
αGC C20:2
5.9%
18.2%
24.2%
b
IFN-γPos NKT
IFN-γPos NK
Cells (X 10³)
c
CD69 MFI (NKT)
CD25 MFI (NKT)
IL-4 (pg/ml)
IFN-γ (ng/ml)
d
IL-2 (ng/ml)
Cells (X 1000)
e
IL-4 (ng/ml)
Glycolipid (nM)

Figure S4

Figure S4, related to Figure 4: Functional analysis of αGC loaded CD1d complexes on different splenic cell types after αGC administration. **(a)** Mice were injected i.p. with the indicated glycolipids and spleens were harvested at 16 hrs post injection for αGC C26:0 (top) or 2 hours post injection for αGC C20:2 (bottom). $CD19^{Pos}$ cells, $CD8\alpha^{Pos}$ DCs and $CD8\alpha^{Neg}$ DCs were purified, and then cultured with iNKT cell enriched (CD19 and CD11c depleted) splenocytes for 16 hours. Plots show representative data for intracellular IFN-γ staining on tetramer positive iNKT cells, cultured with 3 x 10^5 of each of the different purified APC populations. **(b)** Same analysis as described in (a) showing IFN-γ positive cells from cultures with various numbers of APCs. Symbols indicate cultures containing $CD8\alpha^{Pos}$ DCs (black squares) , $CD8\alpha^{Neg}$ DCs (white squares) and CD19Pos cells (black triangles). Responses of both iNKT cells ($NK1.1^{Pos}$ Tet^{Pos}, top) and NK cells ($NK1.1^{Pos}$ Tet^{Neg}, bottom) cells are shown to APCs from animals injected with αGC C26:0 (left) or αGC C20:2 (right). **(c)** For cultures described in (a), CD69 and CD25 expression on iNKT cells ($NK1.1^{Pos}$ Tet^{Pos}) was analyzed, and culture supernatants were collected after 24 hours for measurement of levels of secreted IL-4 and IFN-γ. **(d)** Isolated APCs from glycolipid treated mice as in (a) were cultured with iNKT cell hybridoma DN32.D3, and supernatants were harvested 24 hours later for measurement of IL-2 secretion. Symbols as in (b). **(e)** Effect of depletion of $CD11b^{Pos}$ cells from splenic suspensions on APC function. Immunomagnetic depletion of $CD11b^{Pos}$ cells (filled symbols) or mock depletion (open symbols) was carried out on spleen cell suspensions. Resulting cells (2 x 10^5 per well) were cultured with various concentrations of αGC C26:0 and αGC C20:2 for 16 hrs, and supernatants were harvested for measurement of secreted IFN-γ and IL-4 levels by ELISA. Data shown are means and S.D.

Figure S5

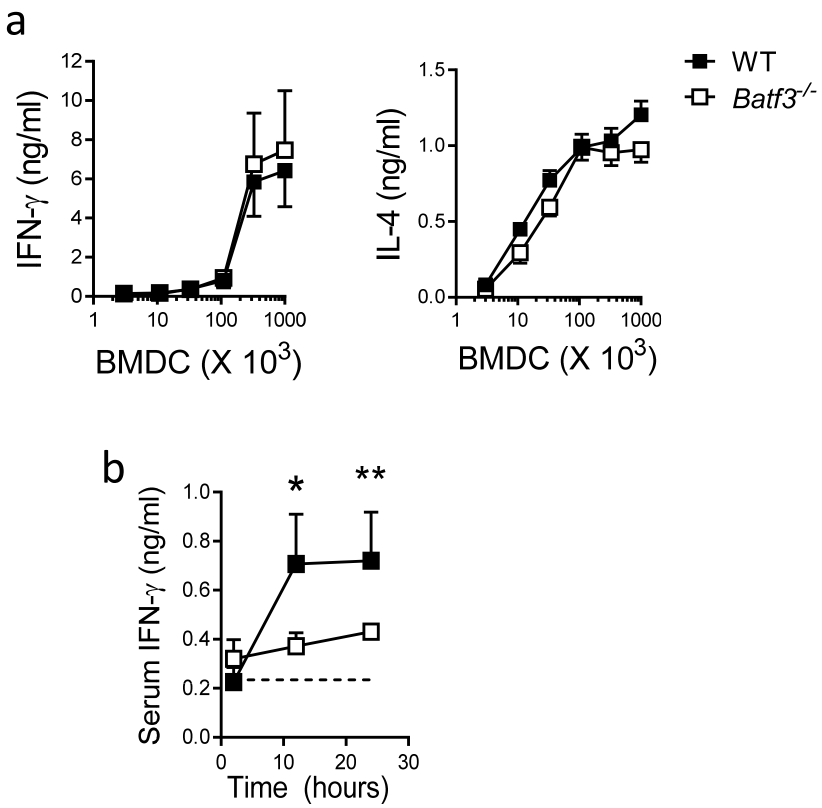

Figure S5, related to Figure 5: (a) Various numbers of bone marrow derived dendritic cells (BMDC) derived from WT mice were pulsed with 100nM of αGC C26:0 for 12 hrs. Cells were washed extensively and co-cultured with either WT or *Batf3*$^{-/-}$ splenocytes for another 24 hours. Supernatants were analyzed for secreted IFN-γ and IL-4 by ELISA. Results are means and S.D. for groups of 3 mice. **(b)** Serum IFN-γ analyzed at various times after i.p. injection of 10 nanomoles of β-GluCer C24:1, a candidate self glycolipid antigen, into WT (filled symbols) and *Batf3*$^{-/-}$ (open symbols) mice. Dashed line indicates level of IFN-γ in mice injected with vehicle only. Results are means and S.D. for groups of 4 mice; $^{*}P < 0.05$, $^{**}P < 0.01$ (2-way ANOVA, Tukey post test). IL-4 was not detected in the sera of these animals at any time point.

Figure S6

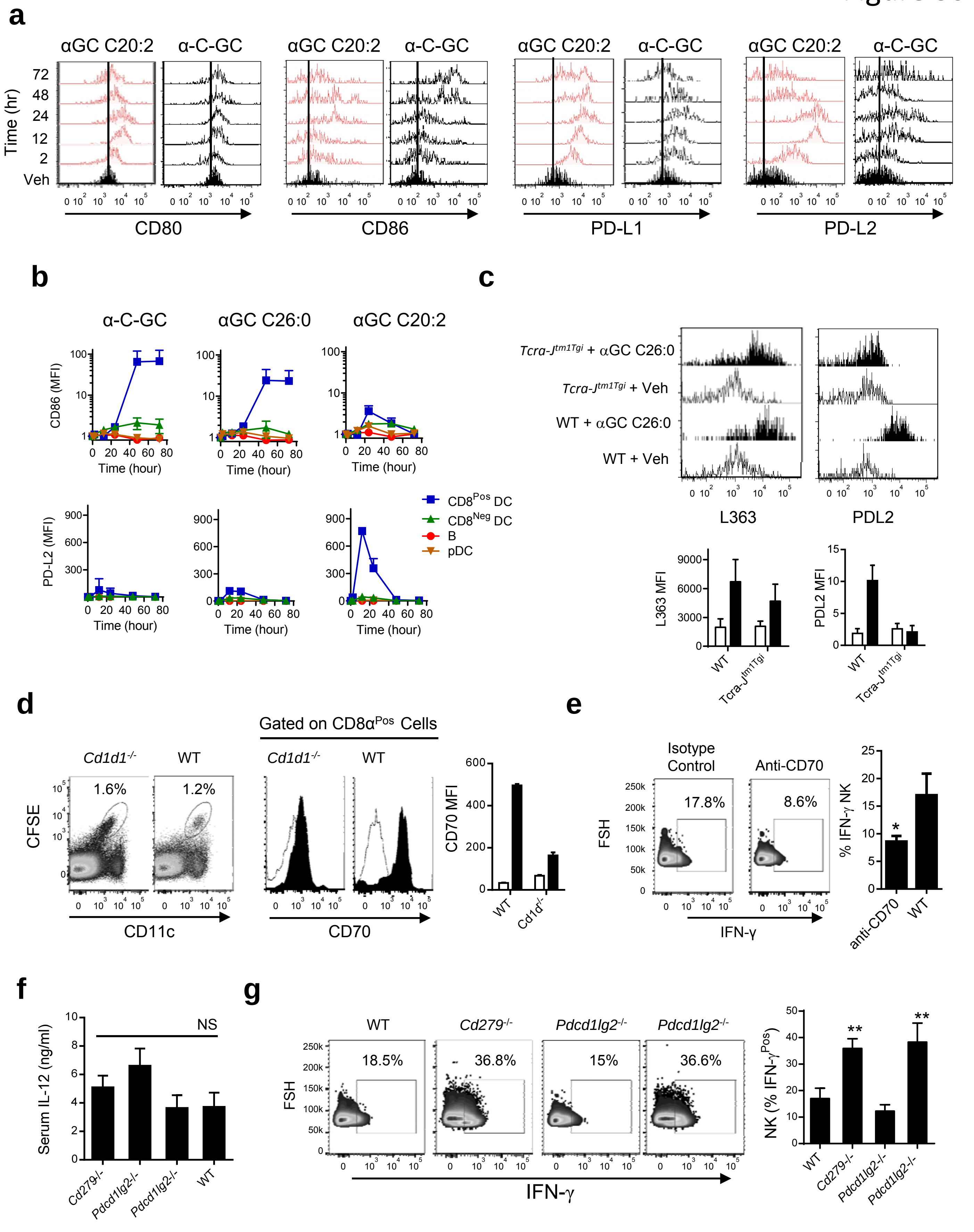

a
αGC C20:2
α-C-GC
Time (hr)
72
48
24
12
2
Veh
CD80
CD86
PD-L1
PD-L2
b
α-C-GC
αGC C26:0
αGC C20:2
CD86 (MFI)
PD-L2 (MFI)
Time (hour)
CD8Pos DC
CD8Neg DC
B
pDC
c
Tcra-Jtm1Tgi + αGC C26:0
Tcra-Jtm1Tgi + Veh
WT + αGC C26:0
WT + Veh
L363
PDL2
L363 MFI
PDL2 MFI
WT
Tcra-Jtm1Tgi
d
Gated on CD8αPos Cells
Cd1d1-/-
WT
1.6%
1.2%
CFSE
CD11c
CD70
CD70 MFI
Cd1d-/-
e
Isotype Control
Anti-CD70
17.8%
8.6%
FSH
IFN-γ
% IFN-γ NK
anti-CD70
f
NS
Serum IL-12 (ng/ml)
Cd279-/-
Pdcd1lg2-/-
g
18.5%
36.8%
15%
36.6%
NK (% IFN-γPos)

Figure S6, related to Figure 6: Selective effects of different glycolipid antigens on costimulatory and co-inhibitory molecule expression by CD8α^{Pos} DCs, and role of CD1d. **(a)** Flow cytometric data for the changes in the expression of CD80, CD86, PD-L1 and PD-L2 on CD8α^{Pos} DCs at various times post α-C-GC(open black histograms) and αGC C20:2 (open red histograms) glycolipid administration. Mice (C57BL/6) were injected i.p. with 2 nanomoles of glycolipids, and splenocytes were obtained for flow cytometry at the indicated times. CD8α^{Pos} DCs were gated as shown in Supplementary Figure 1. Black filled histograms show staining of CD8α^{Pos} DCs from vehicle treated control mice. **(b)** Selective upregulation of CD86 and PD-L2 by CD8α^{Pos} DCs. C57BL/6 mice were treated with 2 nanomoles of the indicated αGC analogues and spleen cells were harvested at different time points. Changes in levels of surface expression of CD86 and PD-L2 on B cells (B220^{Pos}CD11c^{Neg}), pDCs (B220^{Pos} CD11c^{Low}), CD8α^{Neg} DCs (CD11c^{Hi} CD8α^{Neg}) and CD8α^{Pos} DCs (CD11c^{Hi} and CD8α^{Pos}) are shown. Data for each time point are shown as mean fluorescence intensity for groups of 3 mice. **(c)** WT and iNKT deficient *Tcra-J^{tm1Tgi}* (Jα18$^{-/-}$) mice were injected i.p. with 2 nanomoles of αGC C20:2. Splenocytes were harvested after 12 hr, and levels of L363 and anti-PD-L2 were analyzed on CD11c^{Pos} CD8α^{Pos} cells by flow cytometry. **(d)** Flow cytometric data showing gating strategy for CFSE labelled CD11c^{Pos} cells transferred from WT or *Cd1d1$^{-/-}$* animals into WT hosts. Histograms show increase in CD70 staining in αGC C26:0 (2 nanomoles i.p.) treated mice (black) over that of isotype control antibody (white). A bar graph showing mean with range of MFI for CD70 staining of duplicate samples is shown on the right. **(e)** B220^{Pos} and dead cells were excluded before gating on the NK1.1 and NKp46 double positive (NK) cells . Plots show the percentage of IFN-γ positive gated NK cells in response to either isotype control or anti-CD70 treated mice injected with αGC C26:0 (2 nanomoles i.p.). Percentage of IFN-γ positive NK cells for groups of mice (N = 3) is shown in the graph. **(f)** Peak levels of serum IL-12 following i.p. injection of αGC C26:0 (2 nanomoles i.p.) into WT or mice genetically ablated of PD-1, PD-L1 or PD-L2 (*Cd279$^{-/-}$*, *Pdcd1lg2$^{-/-}$* , *Pdcd1lg2$^{-/-}$*). **(g)** FACS analysis of NK cells showing the percentage of IFN-γ positive cells in WT or mice genetically ablated of PD-1, PD-L1 or PD-L2. *P < 0.05, **P < 0.01 (Student's t test for comparisons of two groups, or one way ANOVA for comparisons of three or more groups with *Dunnett's correction.*). Mean with standard deviation is shown for groups of three or more mice.
